# Microencapsulated essential oils combined with organic acids improves immune antioxidant capacity and intestinal barrier function as well as modulates the hindgut microbial community in piglets

**DOI:** 10.1186/s40104-021-00670-3

**Published:** 2022-02-11

**Authors:** Jiayu Ma, Shenfei Long, Jian Wang, Jie Gao, Xiangshu Piao

**Affiliations:** 1 State Key Laboratory of Animal Nutrition, College of Animal Science and Technology, China Agricultural University, Beijing, 100193 China; 2 Institute of Animal Sciences, Chinese Academy of Agricultural Sciences, Beijing, 100193 China

**Keywords:** Essential oil, Gut microbiota, Intestinal barrier, Mixed organic acid, Piglets

## Abstract

**Background:**

The objective of this experiment was to evaluate the effect of a combination of microencapsulated essential oils and organic acids (MOA) on growth performance, immuno-antioxidant status, intestinal barrier function and microbial structure of the hindgut in piglets. A total of 120 piglets (Duroc × [Landrace × Yorkshire]; weighted 7.66 ± 1.79 kg, weaned at d 28) were randomly selected and allocated to 3 treatments with 4 replicates per group and 10 piglets per replicate according to the initial body weight and gender. The dietary treatments were as follows: 1) basal diet (Ctrl); 2) Ctrl + chlortetracycline (75 mg/kg) (AGP); 3) Ctrl+ MOA (1500 mg/kg). The experiment period was lasted for 21 d.

**Results:**

Compared to the Ctrl group, dietary supplemented MOA alleviated (*P* < 0.05) the diarrhea rate from d 12 to 21, enhanced (*P* < 0.05) the concentration of serum interlukin-10 and glutathione peroxidase in piglets on d 11 after weaning and serum superoxide dismutase in 21-day piglets. The MOA group also improved (*P* < 0.05) the apparent digestibility of dry matter (DM), organic matter (OM) and gross energy (GE), up-regulated (*P* < 0.05) the mRNA expression level of occludin, claudin-1 and mucin-2 in ileum and increased (*P* < 0.05) the contents of propionic and butyric acids in the cecum of piglets. The MOA group modulated the cecal and colonic microbial community structure and increased (*P* < 0.05) the abundance of *Faecalibacterium* and Muribaculaceae in cecum and *Streptococcus* and *Weissella* in colon. Additionally, AGP group decreased (*P* < 0.05) apparent digestibility of DM, OM and GE as well as down-regulated (*P* < 0.05) relative gene expression level of claudin-1 in duodenum and jejunum, *ZO-1* and mucin-1 in jejunum of piglets.

**Conclusion:**

In summary, dietary supplemented MOA alleviated diarrhea and improved nutrient apparent digestibility in piglets via enhancing immuno-antioxidant properties, increasing digestive enzyme activity, up-regulating the expression of intestinal barrier-related genes, and modifying the microbial community structure of the cecum and colon. Therefore, dietary supplementation with MOA as an alternative to antibiotics was feasible to improve intestinal health of piglets in practical production.

## Introduction

Stress triggered by early weaning affects the normal physiological course of intestinal microorganisms, resulting in an imbalance of intestinal microorganisms, which induce the intestinal diseases [1]. For newborn piglets, the weaning period is characterized by impaired growth, increased incidence of diarrhea and other diseases as well as severe morphological alterations of the intestinal tissues [2, 3], which alleviated and improved by antibiotic growth promoters (AGP) consequently. Due to the rapid proliferation of livestock production, feed antibiotics are extensively used in breeding production because of their effectiveness in improving feed utilization, promoting growth and maintaining health of livestock [4]. Nonetheless, the shortcomings of over-use of antibiotics are increasingly prominent, such as lowering the immunity, strengthening the resistance of pathogenic bacteria, disturbing the intestinal flora of livestock and the antibiotic residues [5, 6], which have triggered the extensive concern of consumers worldwide. The European Union [7], the United States [8] and China [9] have prohibited and restricted the application of antibiotics in the breeding industry, which is undoubtedly a gigantic challenge for feed enterprises. Hence, there is an urgent requirement for enterprises to develop a novel type of green and safe products which contains the growth-promoting properties of antibiotics and the advantages of pollution-free, residue-free as well as toxin-free.

With the continuous attempts and explorations of scholars, they found that organic acids, plant essential oils, enzymes and antimicrobial peptides could effectively improve performance with no side effects [10, 11]. Several studies demonstrated that plant essential oils were useful in regulating animal intestinal microorganisms, strengthening immuno-antioxidant properties, and improving growth performance [12, 13]. Mixed organic acids are favored by feed and livestock enterprises for their benefits in improving performance and intestinal health of livestock, which rendered mixed organic acids as the most promising green alternative to antibiotics [14, 15]. However, there is no single feed additive available in feed that can completely replace antibiotics. Xu et al. [16] evaluated the role of antibiotic alternatives by meta-analyses system and the findings indicated that a combination of different alternatives to antibiotics may be the most hopeful way to decrease or substitute antibiotics in animal feed. Also, the lowered pH value of the gastrointestinal tract induced by organic acids favors the strengthening of the antibacterial effect of the essential oils, while weakening the strong odor of the oils themselves [17, 18]. A combination of microencapsulated essential oils and organic acids (MOA) is probably extraordinarily effective. Therefore, the purpose of the experiment was to investigate the effect of the combination of MOA on growth performance, immuno-antioxidant properties and intestinal barrier function in piglets, exceedingly focused on the microbial structure of the hindgut of piglets.

## Materials and methods

### Animal ethics

All the programs performed in our animal experiment were endorsed and authorized by the Institutional Animal Care and Use Committee of China Agricultural University (No.AW10601202–1-2, Beijing, China).

### MOA product and antibiotic

The product of MOA combination named PORCINAT+^TM^ was supplied by Jefo Nutrition Inc. (Jefagro, Saint-Hyacinthe, Canada), which is a selected formulation of essential oils primarily containing thymol, vanillin and eugenol and organic acids mainly containing fumaric, sorbic, malic and citric acid microencapsulated in a triglyceride matrix of hydrogenated vegetable oils. The chlortetracycline was sourced from Beijing Tongli Xingke Agriculture Technology Co., Ltd. (Beijing, China).

### Experimental design and diets

A total of 120 piglets (Duroc × [Landrace × Yorkshire]; weighted 7.66 ± 1.79 kg, weaned at d 28) were selected and randomly allocated to 3 treatments with 4 replicates per group and 10 piglets per replicate according to the initial body weight and gender. The dietary treatment was as follows: 1) corn-soybean meal basal diets (Ctrl); 2) Ctrl + 75 mg/kg chlortetracycline (AGP); 3) Ctrl + 1500 mg/kg MOA (MOA). The experiment period was lasted for 21 d. Table 1 lists the composition and nutritional levels of basal diets, which satisfied or excelled the NRC (2012) [19] requirements.

**Table 1:** Composition and nutrient profile of the basal diets (as fed basis), %

Ingredients	Content	Nutritional level	Content
Corn, CP 7.6%	60.24	Calculated values	
Whey powder, CP 3.8%	10.00	Digestive energy, kcal/kg	3487
Soybean meal, CP 43.6%	13.90	Crude protein	18.50
Fermented soybean meal, CP 51%	5.00	Ether extract	4.20
Angel yeast, CP 51.3%	3.00	Lactose	8.00
Fish meal, CP 65.3%	3.00	Calcium	0.60
Soybean oil	1.00	Phosphorus	0.48
Salt	0.40	Sodium	0.31
Dicalcium phosphate	0.54	Lysine	1.54
Limestone	0.28	Methionine	0.56
L-Lysine HCl, 78%	0.71	Threonine	0.83
DL-Methionine, 98%	0.33	Tryptophan	0.27
Threonine, 98%	0.33		
L-Tryptophan, 98%	0.11	Analyzed values ^2^	
Valine	0.18	Gross energy, kcal/kg	3867
Zinc oxide	0.20	Crude protein	18.42
10,000-IU Phytase	0.03	Ether extract	4.37
Chromic oxide	0.25	Dry matter	87.29
Non-antibiotic premix^1^	0.50	Organic matter	94.80
Total	100.00		

^1^Non-antibiotic premix for per kilogram diet included: vitamin A, 12,000 IU; vitamin D_3_, 2000 IU; vitamin E, 24 IU; vitamin K_3_, 2.0 mg; vitamin B_1_, 2.0 mg; riboflavin, 6.0 mg; vitamin B_6_, 3 mg; vitamin B_12_, 24 μg; nicotinic acid, 30 mg; pantothenic acid, 20 mg; folic acid, 3.6 mg; biotin, 0.1 mg; choline chloride, 0.4 mg; iron, 96 mg; copper, 8.0 mg; zinc, 120 mg; manganese, 40 mg; iodine, 0.56 mg; selenium, 0.4 mg

^2^The analyzed values were the average of the 3 feed nutrient levels measured in the Ctrl antibiotic growth promoter (AGP) and microencapsulated essential oils and organic acids (MOA) groups

The experimental piglets were kept in pens of 1.2 m × 2 m with leaky sprayed plastic floor. Each pen was placed with one 3-hole stainless steel adjustable trough and one nipple type waterer. The piglets were kept in “all-in, all-out” feeding management, and the nursery was thoroughly disinfected and cleaned before the piglets were transferred. The nursery temperature (28 °C to 30 °C for the first week, then lowered by 1 to 2 °C weekly up to 23 °C to 25 °C), humidity (60 to 70%) and CO_2_ concentration (below 0.15%) were controlled by an automatic monitoring system. The piglets were fed in the way of “frequent addition and less feeding”, and the transition was performed in the ratio of 1:3, 3:1 and 2:2 according to the Creep feed:Experimental feed. Moreover, the weaned piglets were vaccinated on d 7, d 14 and d 21, respectively (highly pathogenic blue ear disease weak vaccine, pseudorabies vaccine, swine fever weak freeze-dried vaccine) and dewormed regularly. Feeding and health conditions of piglets were observed and recorded at all times. The piglets were fed with powder and allowed to feed and drink ad libitum overall. No mortality occurred for piglets during the experimental period.

### Sampling collection and detection method

On d 11 and d 21 of the experiment, 10 mL of blood was gathered from the anterior vena cava of piglets into a vacutainer, centrifuged at 3000 × *g* for 15 min, the serum was separated and stored at − 20 °C in 0.5 mL centrifuge tubes for analysis of serum immune function and antioxidant characteristic.

On the d 18 of the experiment, feces of piglets in the nursery were cleaned, and fecal samples in each replicate were gathered from the d 19 to 21, the sample collection twice per day without contamination, then stored at − 20 °C for determination of the apparent digestibility of nutrients. Moreover, approximately 1 kg of representative feed samples were harvested weekly during the experiment.

On d 21 of the experiment, one piglet with mean weight was sampled for slaughter per replicate, the liver was gathered and approximately 1 to 2 cm of intestinal samples were collected from the duodenum, jejunum and ileum at the 1/3 of the posterior segment respectively, the intestinal contents were washed off with 0.9% saline gently, placed in 10-mL cryovials and pre-stored in a liquid nitrogen tank, then transferred to − 80 °C for determination of antioxidant enzyme properties, digestive enzyme activity and the expression of intestinal tight junction protein gene. Meanwhile, the tissues of approximately 2 cm small intestine were gathered, washed with sterile saline and fixed in 4% paraformaldehyde for determining intestinal morphology. Furthermore, the cecum and colon contents of piglets were collected for volatile fatty acid analysis and 16srRNA gene sequencing. The surgical trays, scalpel scissors and other instruments used in the sample collection process and the operating table were disinfected with 75% alcohol.

### Growth performance measurement

Piglets were weighed on d 11 and d 21 of the experiment as well as recorded the feed consumption to calculate average daily gain (ADG), average daily feed intake (ADFI) and feed conversion ratio (FCR = ADFI/ADG). The piglets’ anuses were checked one by one at 09:00 and 17:00 daily during the experiment to observe and recorded any fecal contamination and redness. The number of piglets with diarrhea per treatment was counted at the end of the experiment and the diarrhea rate was calculated with the following formulation:

Diarrhea rate (%) = 100% × total number of piglets with diarrhea/(total number of piglets × number of days).

### Serum biochemical immunity and intestinal enzyme activity

The serum was defrosted at 4 °C and mixed well before analysis. The enzyme activities of malondialdehyde (MDA), superoxide dismutase (SOD), glutathione peroxidase (GSH-Px) and total antioxidant capacity (T-AOC) in serum were analyzed by automatic biochemical analyzer (GF-D200, Gaomi Caihong Analytical Instrument, Co Ltd., Shandong, China). Immunoglobulins (IgA, IgG, IgM), interleukin-1β (IL-1β), interleukin-10 (IL-10), gamma-interferon (IFN-γ) tumor necrosis factor-α (TNF-α), D-lactic acid (DLA) and diamine oxidase (DAO) levels in serum were tested by ELISA with enzyme marker (Multiskan Ascent, Thermo Scientific, Waltham, USA). The activities of small intestinal amylase, lipase, trypsin and chymotrypsin were measured by Immunoturbidimetry. The commercial kits were sourced from Nanjing Jiancheng Institute of Biological Engineering (Nanjing, China).

### RNA extraction and real-time PCR

The total RNA was extracted from small intestine of piglets by means of TRizol method according to the manufacture instruction (Thermo Fisher, Waltham, USA), the concentration and quality of RNA were detected by protein nucleic acid assay (ND-2000UV, Thermo Fisher, Waltham, USA) and 1% agarose gel electrophoresis. The RNA was reverse-transcribed into cDNA using the TransScript All-in-One First-Strand cDNA Synthesis SuperMIX for qPCR kit (QIAGEN, Frankfurt, Germany). The reverse transcription system: total RNA, 0.5 μg; 5 × TransScript All-in-one SuperMix for qPCR, 5 μL; gDNA Remover, 0.5 μL; Nuclease-free H_2_O was added to 10 μL. The reaction procedure: 42 °C for 15 min, 85 °C for 5 s. Addition of 90 μL Nuclease-free H_2_O after reverse transcription, then held at − 20 °C.

Real-time PCR was conducted by a LightCycler® 480 II Real-time PCR Instrument (Roche, Basel, Switzerland) (PCR efficiency: 94 to 105%) with 10 μL of PCR reaction mixture, which included 1 μL of cDNA, 5 μL of 2 × PerfectStartTM Green qPCR SuperMix, 0.2 μL of forward primer, 0.2 μL of reverse primer and 3.6 μL of nuclease-free water. The reactions were incubated in 384-well optical plates (Roche, Basel, Switzerland) for 30 s at 94 °C, followed by 45 cycles of 5 s at 94 °C, 30 s at 60 °C. The melting curve analysis was performed at the end of the PCR cycle to verify the specific generation of the expected PCR product. Triplicate analyses were performed for each sample. The primer sequences shown in Table 2 were designed by Ouyi Biotech (Shanghai, China) and synthesized by TsingKe Biotech (Beijing, China) based on the mRNA sequences available from the NCBI database. The expression levels of mRNAs were normalized to the glyceraldehyde-3-phosphate dehydrogenase (*GAPDH*) and were calculated using the 2^-ΔΔCt^ method.

**Table 2:** Primer sequences of housekeeping and target genes concerned with intestinal barrier function

Item^1^	Sequences (5′ to 3′)^2^	Length, bp	T_m_, °C
Occludin	F: GTGGGACAAGGAACGTATT R: TCTCTCCGCATAGTCCGAA	115	60
Claudin-1	F: ATACAGGAGGGAAGCCAT R: ATATATTTAAGGACCGCCCTCT	89	60
*ZO-1*	F: GCTCAGCCCTATCCATCT R: GGACGGGACCTGCTCATAA	90	60
Mucin-1	F: GTGCCGCTGCCCACAACCTG R: AGCCGGGTACCCCAGACCCA	141	60
Mucin-2	F: CAGACCTACTCAGAGTTCCT R: CTCGGGCTTGTTGATCTT	84	60
*GAPDH*	F: CAGCAATGCCTCCTGTACCA	72	60
	R: ACGATGCCGAAGTTGTCATG		

^1^*ZO-1*, zonula occludens-1; *GAPDH*, glyceraldehyde-3-phosphate dehydrogenase

^2^*F*, forward primer; *R*, Reverse primer

### Apparent total tract digestibility (ATTD) of nutrients

The fecal samples were thawed at 4 °C, then dried at 65 °C for 72 h, the samples of feed and feces were ground through a 40-mesh (425 μm) screen before analysis. Dry matter (DM), crude protein (CP), ether extract (EE) and ash were analyzed in accordance with the Association of Official Analytical Chemists [20]; Neutral detergent fiber (NDF) and Acid detergent fiber (ADF) were determined with reference to the method of Vansoest et al. [21] (A2000i fiber analyzer, Ankom, Macedon, USA). The gross energy in feed and feces samples was analyzed using 6400-Automatic Isoperibol Calorimeter (PARR, Moline, USA). The chromium levels in feed and feces were analyzed by atomic absorption spectrophotometer (Z-5000; Hitachi, Tokyo, Japan) based on the methodology of Williams et al. [22] for calculating the ATTD with the following equation.

Apparent total tract digestibility (ATTD, %) = 1 - (Cr _feed_ × Nutrient _feces_) / (Cr _feces_ × Nutrient _feed_).

### Intestinal morphology

After the small intestine samples were fixed in 4% paraformaldehyde solution for 48 h, the samples were rinsed, excised, and dehydrated with ethanol for 24 h, then paraffin-embedded, sliced in 4 cross-sections and stained with hematoxylin-eosin. Lastly, the samples were morphologically detected by light microscopy (Olympus CX31, Tokyo, Japan), and 10 intact, well-oriented villi-crypt units were measured in each section. The villi height (VH) was evaluated from the top of the villi to the junction of the villi and crypt, the crypt depth (CD) was determined as the depth of the villi invagination, then calculated the ratio of villi height to crypt depth (VH:CD).

### Cecal volatile fatty acids

The cecal contents of piglets were thawed at 4 °C and mixed, approximately 0.5 g of the sample was weighed into a 10-mL centrifuge tube, adding 8 mL of ultrapure water, sonicated in an ice water bath for 30 min (mixing every 10 min), then centrifuged at 15,000 × *g* for 10 min. The supernatant was diluted 50 times with ultrapure water, filtered through a 0.22 mm membrane, transferred to the 2-mL injection vial and analyzed the volatile fatty acid using a high-performance ion chromatography analyzer (ICS-3000, Thermo Scientific, USA).

### Pyrosequencing of 16S rRNA amplicons

The cecum and colon contents were removed from the − 80 °C refrigerator and the total bacterial DNA was extracted in accordance with the manufacturer’s instructions of FastDNA® SPIN for soil kit (MP Biomedicals, Irvine, USA). The integrity and purity of the DNA was determined using 1% agarose gel electrophoresis and a NanoDrop 2000 spectrophotometer (Thermo Scientific, Waltham, USA). The DNA concentration was quantified precisely by Qubit Fluorometer (Thermo Scientific, Waltham, USA), and the samples were diluted to 1 ng/μL with sterile water. The specific primers with marker sequences (338F: 5′-ACTCCTACGGGAGGCAGCAG-3′ and 806R: 5′-GGACTACHVGGGTWTCTAAT-3′) were employed for PCR amplification of the variable region of 16S rRNA gene V3-V4. The total volume of PCR reaction system is 30 μL, consisting of Phusion® High-Fidelity PCR Master Mix with GC Buffer 15 μL, Phusion® High-Fidelity DNA polymerase 0.5 μL (New England Biolabs, Herts Hitchin, UK), 0.2 μmol/L upstream and downstream 1 μL each, 10 ng/μL genomic DNA 2 μL as well as 10.5 μL of sterilized ultrapure water. The amplification procedure was performed as follows: 98 °C pre-denaturation for 1 min, 98 °C denaturation for 10 s, 50 °C annealing for 30 s, 72 °C extension for 30 s totaling 30 cycles, 72 °C stable extension for 5 min, and lastly stored at 4 °C (PCR instrument: ABI GeneAmp® Model 9700, Applied Biosystems, Foster City, USA).

The PCR products from the samples were mixed and examined by 2% agarose gel electrophoresis; then quantified by Quantus™ Fluor-ST Fluorescence Quantification System (Promega, Madison, USA). The PCR products were mixed in equal amounts following the concentration of the obtained PCR products, re-electrophoresed on 2% agarose gels, the target product bands were recovered using QIAquick Gel Extraction Kit (Axygen, Santa Clara Valley, USA). The library construction was performed by TruSeq® DNA PCR-Free DNA library kit (Illumina, San Diego, USA), and sequenced by HiSeq2500 PE250 after Qubit and qPCR quality control. The raw tags were obtained by splicing the sample reads with FLASH software (http://www.cbcb.umd.edu/software/flash, V1.2.10); the raw tags were filtered and processed to obtain the high-quality effective tags by following the tags quality control process of QIIME [23]. a) truncate the raw tags from the first low-quality site where the number of consecutive low-quality bases (≤ 19) reaches a set length of three; b) filter out the tags with consecutive high-quality bases less than 75% of the tags length from the intercepted tags dataset. The sequences were OTU (operational taxonomic unit) clustered and chimeras were removed based on 97% similarity [24] using UPARSE [25] software (http://drive5.com/uparse/, V7.1) to generate OTU. The RDP classifier [26] (http://sourceforge.net/projects/rdp-classifier/, V2.2) Bayesian algorithm was employed to annotate the OTU sequences against the Silva 16S rRNA database (http://www.arb-silva.de, V138) for species classification (comparison threshold of 70%) and the community composition of each sample at each taxonomic level was counted.

### Statistical analysis

All data were initially organized by Excel software and then statistically analyzed using the GLM model data in SAS software (V9.2, 2008). The dietary treatment as a fixed effect and the block as a random effect. The replicate as a unit to analyze the data concerning growth performance and apparent digestibility of nutrients in piglets, the diarrhea rate of piglets was analyzed by the chi-square test. The data on piglet serum, digestive enzymes, intestinal morphology and so on were analyzed based on the individual as a unit. Multiple comparisons and significance of differences among groups were performed using the Turkey method, means and standard errors were calculated using LSMEANS method; *P* < 0.05 was regarded as statistically significant, 0.05 < *P* ≤ 0.10 was indicative of a differential trend.

For microbiota profiling, statistical analysis of α-diversity, including the Shannon and Simpson indices reflecting microbial diversity and the Chao and ACE indices indicating bacterial abundance [27], was performed by mothur (http://www.mothur.org/wiki/, V1.30.1). The Circos-0.67-7 software (http://circos.ca/) was employed to analyze the relationship between samples and species by visualizing circle plots. The Unifrac distance was computed based on the species differences between the sample sequences and calculated the β-diversity distance by QIIME (http://qiime.org/), the principal coordinates analysis (PCoA) and partial least squares discriminant analysis (PLS-DA) were conducted using R software (V3.4.4). The analysis of between-treatment variance (One-way ANOVA) of α-diversity and β-diversity was performed using R software. The Analysis of similarities (ANOSIM) was used to compare the significance of differences in community structure between treatments. The LEfSe tool (Linear discriminant analysis [LDA] and effect size) was applied to analyze the core flora (LDA score > 3.0) in the cecum and colon of piglets (http://huttenhower.sph.harvard.edu/galaxy/root?tool_id=lefse_upload). The intergroup species differences were analyzed at each taxonomic level using R software based on the obtained bacterial community abundance data and plotted with the “vegan” and “ggplot2” packages.

## Results

### Growth performance

No significant difference was noted on growth performance among treatments with exception of the improved (*P* < 0.05) diarrhea rate of piglets supplemented MOA from d 12 to 21 (Table 3).

**Table 3:** Growth performance of piglets as affected by dietary AGP and MOA supplementation^1^

Item	Ctrl	AGP	MOA	SEM	*P*-value
Day 1 to 11					
ADG, g	373	385	369	18.47	0.82
ADFI, g	532	499	485	26.96	0.51
FCR	1.42	1.28	1.32	0.06	0.28
Diarrhea rate, %	30.53	28.11	23.56	3.18	0.38
Day 12 to 21					
ADG, g	417	455	452	19.01	0.38
ADFI, g	727	751	725	35.37	0.85
FCR	1.74	1.64	1.61	0.05	0.21
Diarrhea rate, %	16.88^a^	16.04^a^	9.79^b^	0.71	0.01
Day 1 to 21					
ADG, g	394	415	406	17.74	0.73
ADFI, g	614	605	586	29.52	0.80
FCR	1.55	1.45	1.45	0.04	0.23
Diarrhea rate, %	24.78	23.03	17.36	1.99	0.12

*ADG* average daily gain, *ADFI* average daily feed intake, *FCR* feed conversion ratio

^a, b^Means in the same row with different superscripts are significantly different (*P* < 0.05)

^1^Control (*Ctrl*): a corn soybean meal-based diet. *AG*P: Ctrl + 75 mg/kg chlortetracycline. *MOA*: Ctrl + 1500 mg/kg MOA. *n* = 4

### Serum immune antioxidant status

Compared to the Ctrl and AGP group, dietary supplementation with MOA enhanced (*P* < 0.05) the concentration of serum IL-10 and GSH-Px of 11d-piglets Also, compared to the AGP, an increased (*P* < 0.05) content of serum T-AOC was observed in 11-day piglets supplemented with MOA (Table 4).

**Table 4:** Serum immune function and antioxidant status of piglets as affected by dietary AGP and MOA supplementation ^1^

Item	Ctrl	AGP	MOA	SEM	*P*-value
Day 11					
IgA, μg/mL	19.58	19.51	19.74	0.72	0.97
IgG, mg/mL	9.16	9.07	9.23	0.41	0.96
IgM, μg/mL	7.06	7.10	8.05	0.34	0.18
IL-1β, ng/L	92.68	94.21	92.86	1.71	0.80
IL-10, ng/L	17.71^b^	17.83^b^	21.69^a^	0.61	0.02
IFN-γ, pg/mL	196.74	197.34	195.90	6.75	0.98
TNF-α, ng/L	56.78	58.79	56.88	1.02	0.38
GSH-Px, μmol/L	8.09^b^	12.24^ab^	15.50^a^	0.83	0.01
SOD, U/mL	109.56	124.03	129.65	4.36	0.07
T-AOC, U/mL	10.41^a^	7.83^b^	11.16^a^	0.45	0.01
MDA, nmol/mL	1.48	1.46	1.30	0.10	0.46
Day 21					
IgA, μg/mL	18.04	19.26	18.64	0.54	0.37
IgG, ng/L	8.57	8.22	8.60	0.14	0.22
IgM, μg/mL	7.17	7.91	8.07	0.21	0.07
IL-1β, ng/L	102.81	101.70	103.90	2.19	0.79
IL-10, ng/L	17.19	18.62	19.57	0.47	0.06
IFN-γ, pg/mL	194.25	201.16	200.61	6.34	0.71
TNF-α, ng/L	50.63	50.88	48.65	1.05	0.36
GSH-Px, μmol/L	13.05	15.46	17.00	1.05	0.13
SOD, U/mL	125.66^b^	143.28^b^	169.51^a^	5.08	0.01
T-AOC, U/mL	10.46	10.62	10.93	0.72	0.90
MDA, nmol/mL	1.37	1.53	1.27	0.11	0.32

*IgA*, *IgG*, *IgM*, immunoglobulin A, G, M; *IL-1β*, *IL-10*, interleukin-1β, 10; *IFN-γ*, gamma-interferon; *TNF-α*, tumor necrosis factor-α; *T-AOC*, total antioxidant capacity; *MDA*, malondialdehyde; *GSH-Px*, glutathione peroxidase; *SOD*, serum superoxide dismutase

^a,b^Means in the same row with different superscripts are significantly different (*P* < 0.05)

^1^Control (*Ctrl*): a corn soybean meal-based diet. *AGP*: Ctrl + 75 mg/kg chlortetracycline. *MOA*: Ctrl + 1500 mg/kg MOA. *n* = 4

Compared to the Ctrl, the concentration of serum IgM (*P* = 0.07) and IL-10 (*P* = 0.06) in 21d-piglets supplemented with MOA showed a tendency to enhance and a higher level (*P* < 0.05) of SOD was observed in 21-day piglets supplemented with MOA.

### Apparent nutritional digestibility

The apparent digestibility of DM, OM and GE in weaning pig dietary supplementation with MOA was outperform (*P* < 0.05) than Ctrl and AGP group. Nevertheless, no significance differences were noticed among treatments on CP, EE, NDF and ADF (Table 5).

**Table 5:** Apparent total tract digestibility of dietary nutrients in piglets at 21 days of age as affected by dietary AGP and MOA supplementation^1^, %

Item	Ctrl	AGP	MOA	SEM	*P*-value
DM	81.25^b^	78.30^c^	83.09^a^	0.29	0.01
OM	84.46^b^	82.05^c^	86.12^a^	0.26	0.01
CP	74.40	70.72	75.79	1.66	0.20
EE	68.93	58.57	68.68	3.86	0.21
GE	79.18^b^	76.52^c^	81.02^a^	0.35	0.01
NDF	52.04	50.78	47.62	2.06	0.39
ADF	26.68	30.11	35.88	4.23	0.39

*DM* dry matter, *OM* organic matter, *CP* crude protein, *EE* ether extract, *GE* gross energy, *NDF* neutral detergent fiber, *ADF* acid detergent fiber

^a,b^Means in the same row with different superscripts are significantly different (*P* < 0.05)

^1^Control (*Ctrl*): a corn soybean meal-based diet. *AGP*: Ctrl + 75 mg/kg chlortetracycline. *MOA*: Ctrl + 1500 mg/kg MOA. *n* = 4

### Digestive enzyme activity

**Fig. 1:**
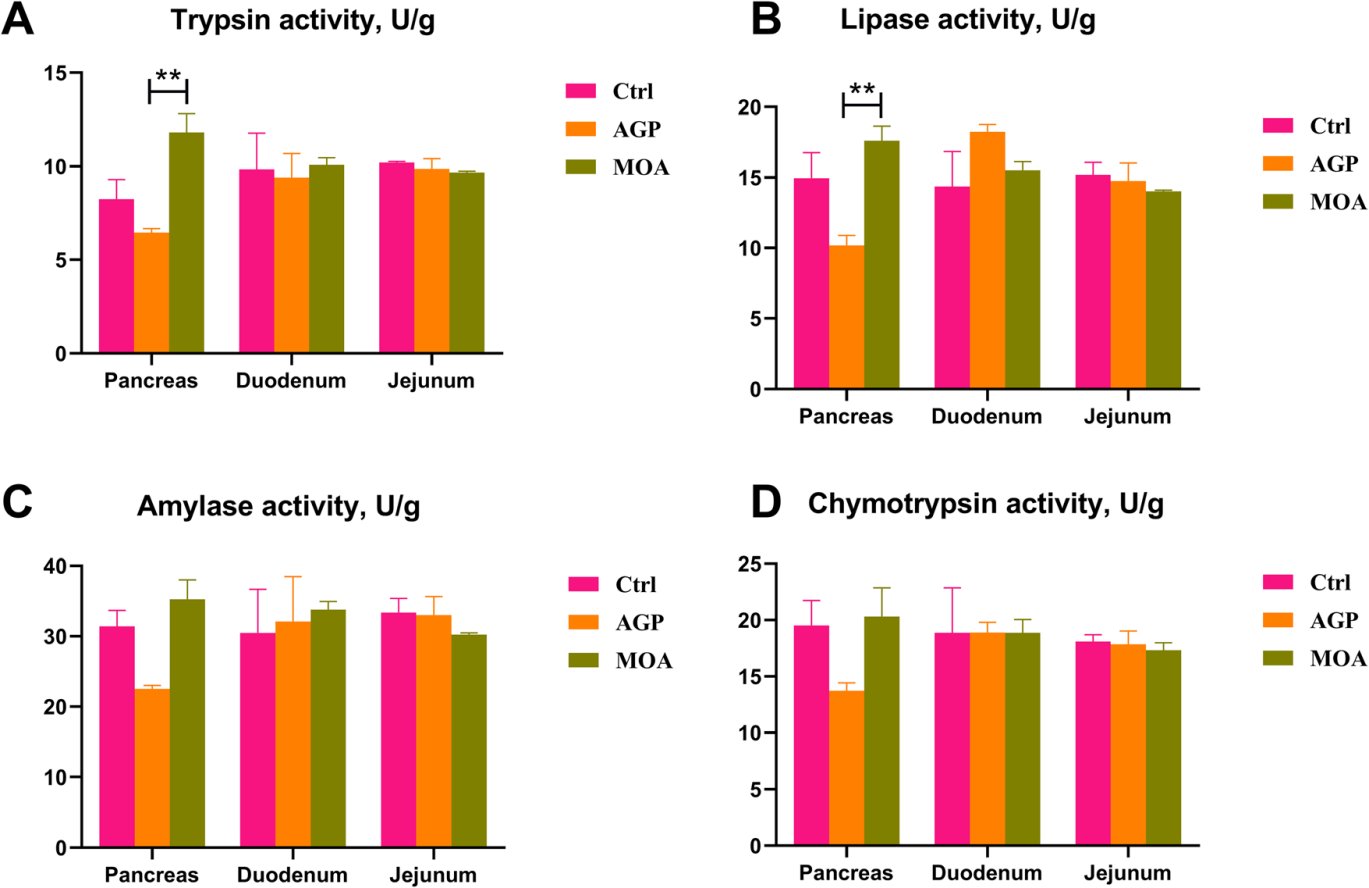
The digestive enzyme activity of pancreas, duodenum and jejunum in piglets at 21 days of age as affected by dietary AGP and MOA supplementation. (A) Trypsin activity. (B) Lipase activity. (C) Amylase activity. (D) Chymotrypsin activity. Control (Ctrl): a corn soybean meal-based diet. AGP: a basic diet with 75 mg/kg of chlortetracycline. MOA: a basic diet with 1500 mg/kg of MOA. Values are indicated as means ± SEM. Bar with the asterisk (*) level suggested the degree of significant difference (* 0.01 < *P* < 0.05, ** 0.001 < *P* < 0.01). *n* = 4

An increased (*P* < 0.05) of trypsin and lipase activities in the pancreas of piglets supplemented with MOA were observed compared to the AGP group (Fig. 1). Nevertheless, compared to the Ctrl, no significant differences were noticed in trypsin, lipase, amylase and chymotrypsin activities in the pancreas, duodenum and jejunum of piglets supplemented with MOA.

### Antioxidant enzyme properties of liver and intestine

**Fig. 2:**
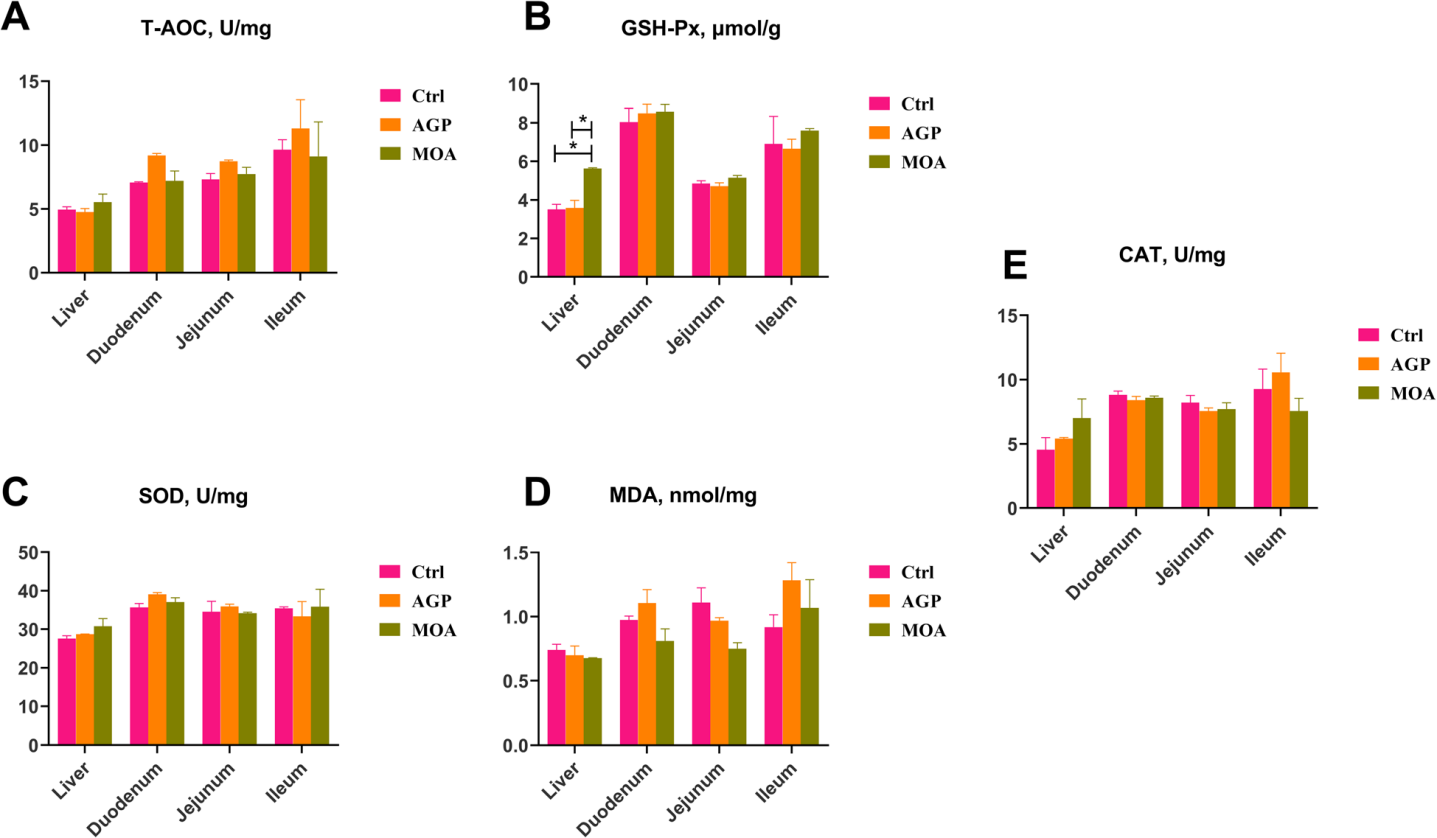
The antioxidant enzyme properties of liver and small intestine in piglets at 21 days of age as affected by dietary AGP and MOA supplementation. (A) Total antioxidant capacity (T-AOC). (B) Glutathione peroxidase activity (GSH-Px). (C) Superoxide dismutase activity (SOD). (D) Malondialdehyde (MDA). (E) Catalase activity (CAT). Control (Ctrl): a corn soybean meal-based diet. AGP: Ctrl + 75 mg/kg chlortetracycline. MOA: Ctrl + 1500 mg/kg MOA. Values are indicated as means ± SEM. Bar with the asterisk (*) level suggested the degree of significant difference (* 0.01 < *P* < 0.05). *n* = 4

Dietary supplementation with MOA enhanced (*P* < 0.05) the level of GSH-Px in liver of piglets (Fig. 2). However, there were no significant differences in T-AOC, SOD, MDA and CAT among the liver, duodenum, jejunum and ileum of piglets supplemented with MOA compared to the ctrl and AGP groups.

### Relative mRNA expression involved in intestinal barrier function

**Fig. 3:**
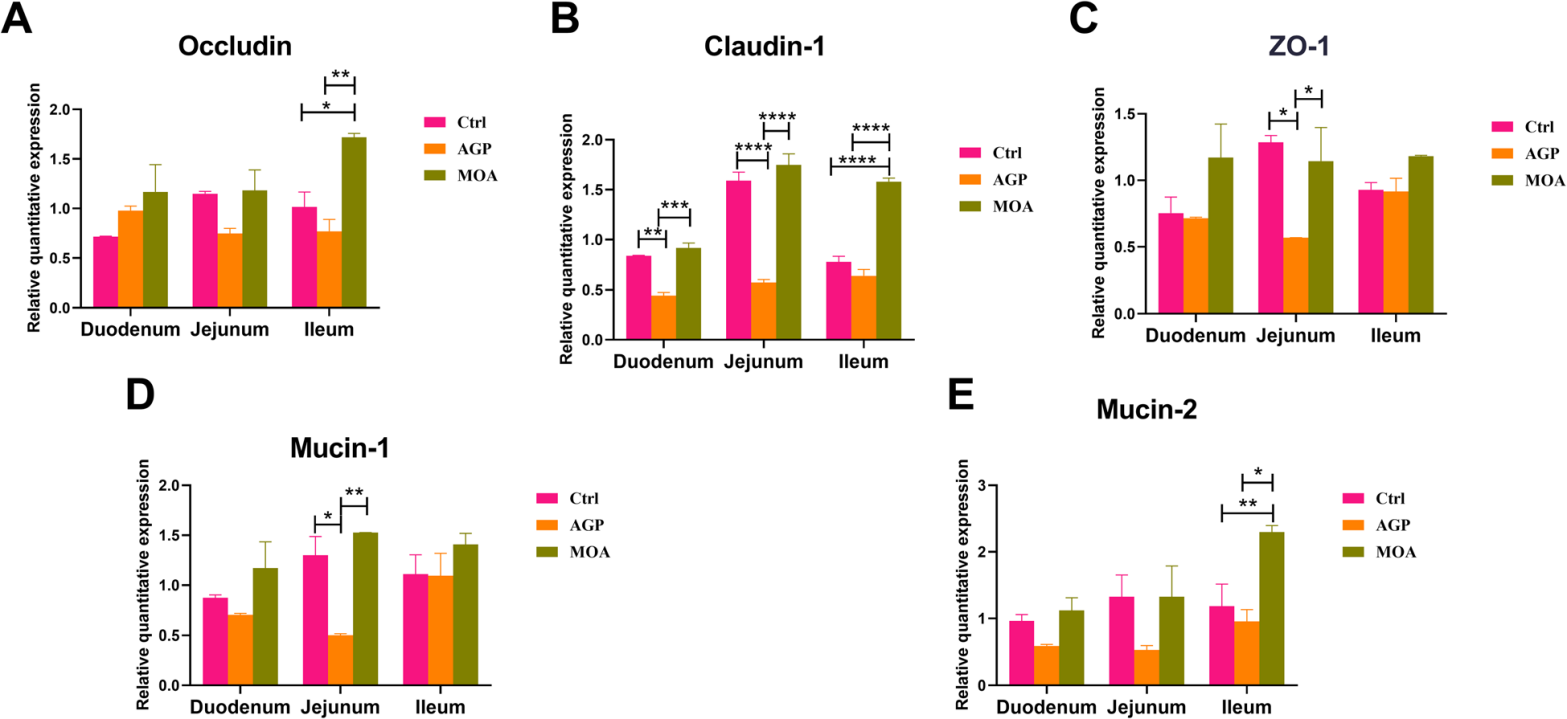
The gene expression involved in intestinal barrier function of small intestine in piglets at 21 days of age as affected by dietary AGP and MOA supplementation. (A) Occludin. (B) Claudin-1. (C) Zonula occludens-1 (*ZO-1*). (D) Mucin-1. (E) Mucin-2. Control (Ctrl): a corn soybean meal-based diet. AGP: Ctrl + 75 mg/kg chlortetracycline. MOA: Ctrl + 1500 mg/kg MOA. Values are indicated as means ± SEM. Bar with the asterisk (*) level suggested the degree of significant difference (* 0.01 < *P* < 0.05, ** 0.001 < *P* < 0.01, *** 0.0001 < *P* < 0.001, **** *P* < 0.0001). *n* = 4

A down-regulated (*P* < 0.05) relative mRNA expression level of claudin-1 in duodenum and jejunum, *ZO-1* and mucin-1 in jejunum were noted in piglets supplemented AGP compared to Ctrl and MOA group (Fig. 3). Also, the relative mRNA expression levels of occludin, caudin-1 and mucin-2 in ileum of piglets supplemented MOA were superior (*P* < 0.05) to Ctrl and AGP group, respectively.

### Intestinal morphology

**Fig. 4:**
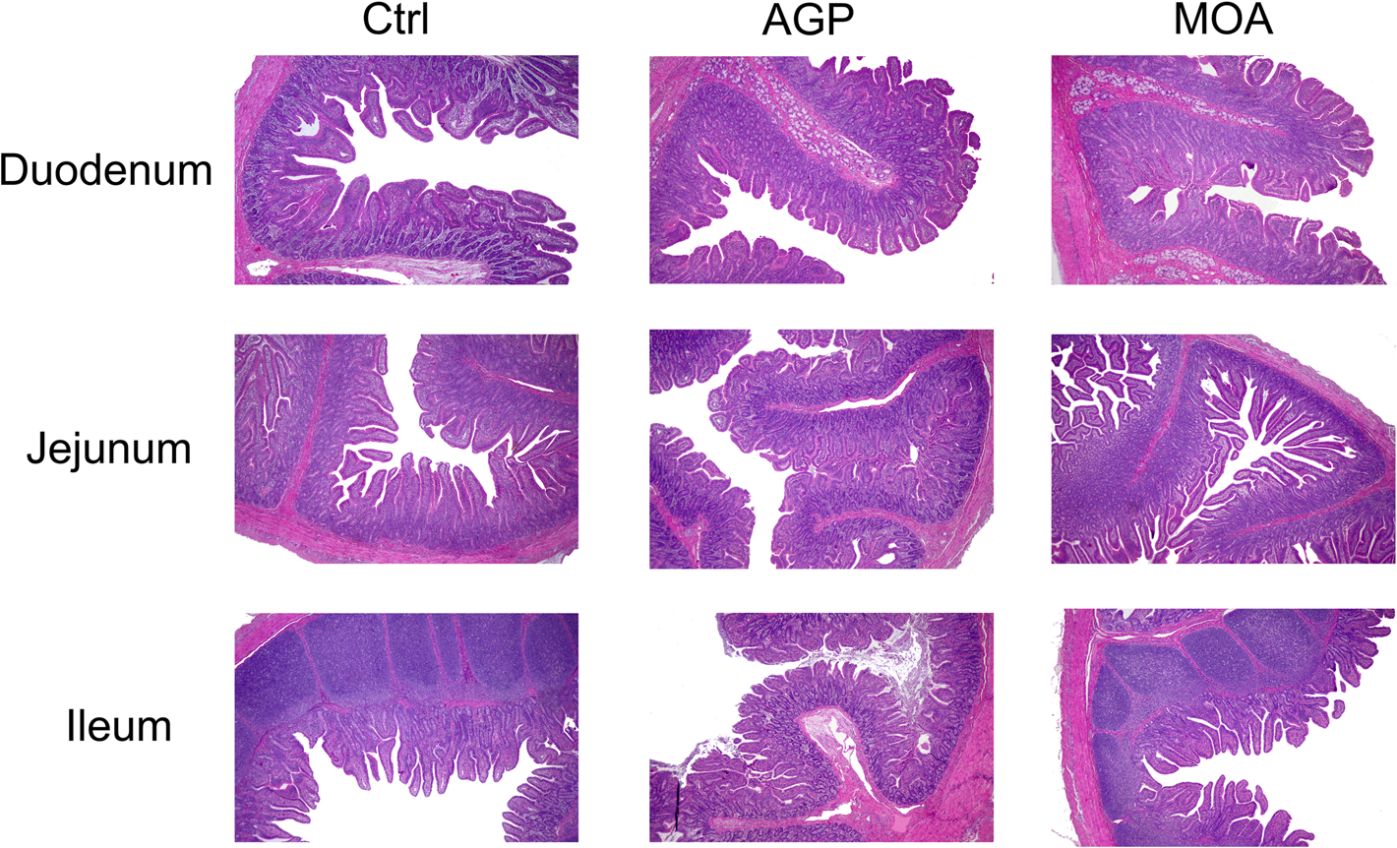
The photomicrograph of small intestinal segments from piglets at 21 days of age. Intestinal morphology in duodenum, jejunum and ileum of piglets as affected by dietary AGP and MOA supplementation. Picutures were observed at 100 × magnification. Control (Ctrl): a corn soybean meal-based diet. AGP: Ctrl + 75 mg/kg chlortetracycline. MOA: Ctrl + 1500 mg/kg MOA. *n* = 4

There was no significant difference occurred on villus height, crypt depth and VH/CD in duodenum, jejunum and ileum of piglets among treatments (Table 6**,** Fig. 4).

**Table 6:** Intestinal morphology of piglets at 21 days of age as affected by dietary AGP and MOA supplementation^1^

Item	Ctrl	AGP	MOA	SEM	*P*-value
Duodenum					
VH, μm	310.18	288.90	325.98	17.76	0.48
CD, μm	209.37	188.79	199.91	5.76	0.24
VH:CD	1.49	1.53	1.64	0.13	0.75
Jejunum					
VH, μm	332.93	330.83	349.62	13.94	0.65
CD, μm	202.92	204.89	208.05	7.05	0.88
VH:CD	1.64	1.61	1.68	0.02	0.16
Ileum					
VH, μm	235.49	234.18	256.52	6.16	0.19
CD, μm	145.10	149.68	159.82	5.59	0.36
VH:CD	1.63	1.57	1.61	0.10	0.92

*VH* Villus height, *CD* Crypt depth, *VH:CD* Villus height to crypt depth ratio

^a,b^Means in the same row with different superscripts are significantly different (*P* < 0.05)

^1^Control (*Ctrl*): a corn soybean meal-based diet. *AGP*: Ctrl + 75 mg/kg chlortetracycline. *MOA*: Ctrl + 1500 mg/kg MOA. *n* = 4

### Volatile fatty acid

The 11-day weaning pig supplemented with MOA increased (*P* < 0.05) the cecal content of propionic acid and butyric acid compared to the Ctrl group (Table 7).

**Table 7:** Volatile fatty acid of cecal contents in piglets at 21 days of age as affected by dietary AGP and MOA supplementation^1^, mg/kg

Items^1^	Ctrl	AGP	MOA	SEM	*P*-value
Day 11					
Lactic acid	0.05	0.30	0.16	0.06	0.10
Acetic acid	2.39	2.13	1.53	0.22	0.11
Propionic acid	1.62^b^	2.31^ab^	2.76^a^	0.17	0.02
Isobutyric acid	0.10	0.11	0.13	0.02	0.64
Butyric acid	1.31^b^	1.62^b^	2.61^a^	0.18	0.01
Isovaleric acid	0.09	0.09	0.13	0.03	0.47
Valeric acid	0.17	0.17	0.13	0.04	0.65
Total volatile fatty acid	5.74	6.73	7.44	0.48	0.15
Day 21					
Lactic acid	0.31	0.54	0.47	0.13	0.50
Acetic acid	3.69	3.20	3.94	0.30	0.32
Propionic acid	2.00^b^	1.55^b^	2.66^a^	0.13	0.01
Isobutyric acid	0.25^a^	0.03^b^	0.04^b^	0.01	0.01
Butyric acid	1.39	1.42	1.90	0.16	0.15
Isovaleric acid	0.21^a^	0.13^b^	0.13^b^	0.01	0.02
Valeric acid	0.38	0.23	0.22	0.07	0.28
Total volatile fatty acid	8.24^ab^	7.10^b^	9.36^a^	0.30	0.02

^a,b^Means in the same row with different superscripts are significantly different (*P* < 0.05)

^1^Control (*Ctrl*): a corn soybean meal-based diet. *AGP*: Ctrl + 75 mg/kg chlortetracycline. *MOA*: Ctrl + 1500 mg/kg MOA. *n* = 4

Dietary supplementation with MOA enhanced (*P* < 0.05) the cecal concentration of propionic acid and total volatile fatty acid. Also, a lowered (*P* < 0.05) concentration of isobutyric acid and isovaleric acid in cecum of 21-day weaning pig dietary supplementation with MOA and AGP compared to the Ctrl.

### Cecal microbiota

The Venn diagram provided a visual representation of the similarity and overlap of the OTU composition of samples by counting the number of OTU that are common and unique to samples.

**Fig. 5:**
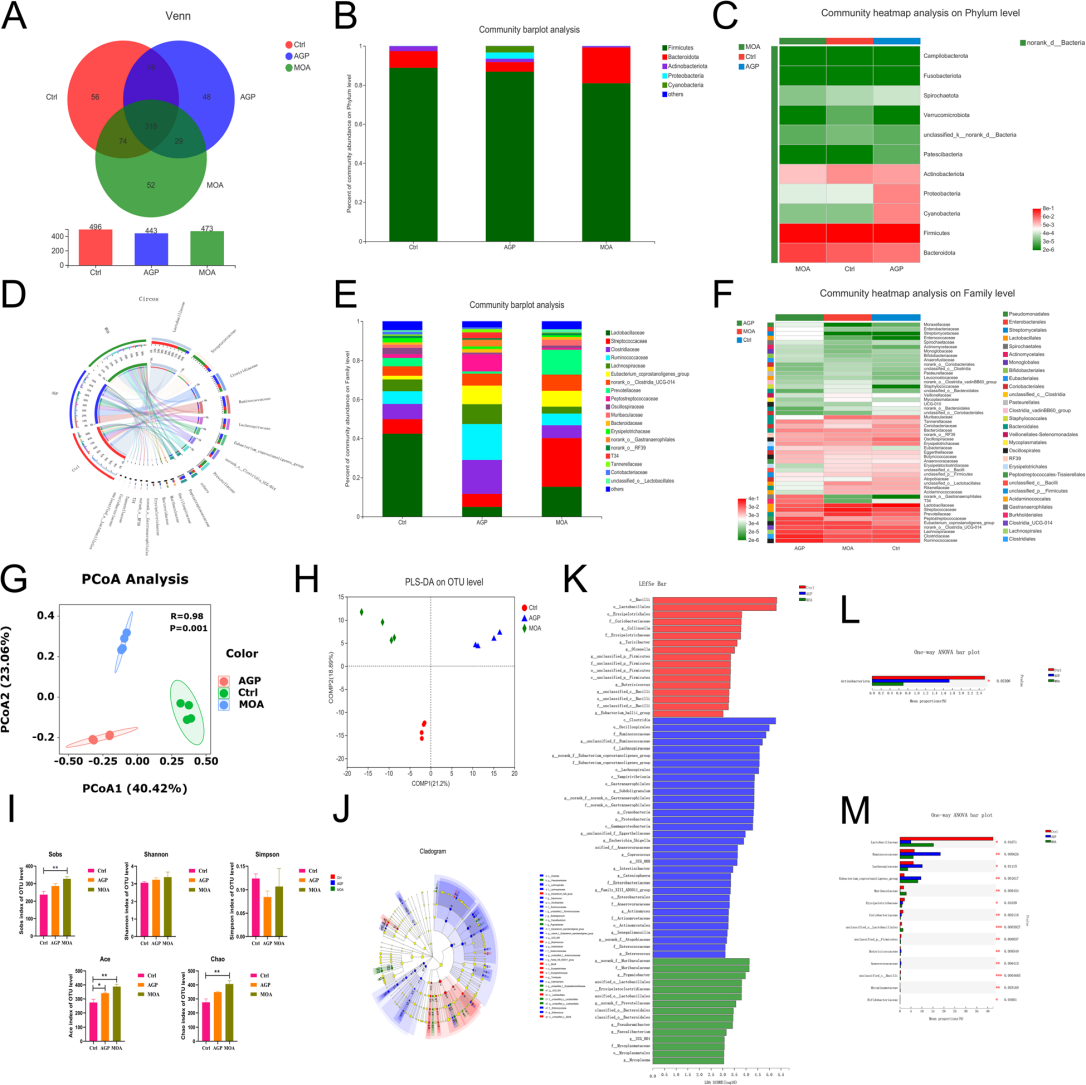
The microbial composition and structure of the cecal contents in piglets as affected by dietary AGP and MOA supplementation. (A) Venn diagram. (B, E) Barplot analysis of microbial community compositions at phylum and family levels. (C, F) Heatmap analysis of microbial community compositions at phylum and family levels. (D) Circos diagram at family level. (G) Principal coordinate analysis (PCoA) based on bray-Curtis distance calculated from OTU abundance matrix (*R* = 0.98, *P* = 0.001). (H) Partial least squares discriminant analysis (PLS-DA) on OTU level, the analysis of similarities (ANOSIM) was used to exam the significant difference between treatments. (I) The α-diversity of microbial community, bar with the asterisk (*) level suggested the degree of significant difference and the values were indicated as means ± SEM (* 0.01 < *P* < 0.05, ** 0.001 < *P* < 0.01). (J) The discriminant analysis of LEfSe multi-level species difference from phylum to genus level. (K) Histogram of linear discriminant analysis (LDA) from phylum to genus level; the values were checked by a non-parametric factorial Kruskal-Wallis rank sum test to identify the microbes with the significant differential characteristics and a linear discriminant analysis was used to assess the degree of impact of abundance on the differences for each species. (L, M) Significance test of difference between treatments at phylum and family levels. Control (Ctrl): a corn soybean meal-based diet. AGP: Ctrl + 75 mg/kg chlortetracycline. MOA: Ctrl + 1500 mg/kg MOA. *n* = 4

From the Venn analysis (Fig. 5A) of OTU in cecum of piglets, which totally acquired 1412 OTU, and 318 were common OTU, the 56, 48 and 52 represented the unique OTU of Ctrl, AGP and MOA, respectively. At the phylum level (Fig. 5B, C), Firmicutes and Bacteroidetes were dominated phylum, which accounting in excess of 90%. The populations of Firmicutes in Ctrl, AGP and MOA group were 88.78%, 86.92% and 81.00%, respectively. The Bacteroidetes in Ctrl, AGP and MOA group were accounting for 8.58%, 4.83% and 18.21%, respectively. At the Family level (Fig. 5E, F), the microorganisms that dominated the top five in the Ctrl group were Lactobacillaceae (42.39%), Streptococcaceae (7.49%), Clostridiaceae (7.80%), Ruminococcaceae (6.53%) and Lachnospiraceae (6.06%). In the AGP group, primarily dominated by Ruminococcaceae (18.42%), Clostridiaceae (17.30%), Lachnospiraceae (10.18%), Eubacterium_coprostanoligenes_group (9.56%) and PeptoStreptococcaceae (8.76%). In the MOA group, mainly dominated by Streptococcaceae (24.89%), Lactobacillaceae (15.27%), Prevotellaceae (12.77%), norank_o_Clostridia UCG-014 (8.30%) and Eubacterium_coprostanoligenes_group (8.14%). The Circos plots (Fig. 5D) reflect the proportional distribution of dominant species in cecum of piglets among the treatments as well as the proportional distribution of each dominant species in the different treatments at the family level. From the PCoA based on Bray_curtis at OTU level (Fig. 5G), the contribution values of the two principal components PC1 and PC2 were PC1 = 40.42% and PC2 = 23.06%, respectively, and the treatments differ in microbial composition (*P* = 0.001, R = 0.98). Similar results were obtained from the PLS-DA plot (Fig. 5H). In α-diversity (Fig. 5I), the Sobs, Chao, and Ace indices were used to reflect the microbial community richness, the Shannon and Simpson indices were applied to mirror the microbial community diversity. The Sobs, Ace and Chao indices in cecum of piglets supplemented with MOA were increased (*P* < 0.05). Also, dietary supplementation with AGP enhanced (*P* < 0.05) the Ace index in cecum of piglets. The microorganisms with significant difference properties were identified by the non-parametric factorial Kruskal-Wallis sum-rank test. The effect of microbial abundance of each species on the difference effect was assessed by LDA (LDA threshold > 3.0). The results (Fig. 5J, K) indicated that the cecal microbiota composition was affected by feeding modification. An increased richness of *Collinsella*, *Turicibacter* and *Olsenella* in Ctrl group, *Subdoligranulum* in AGP group as well as *Faecalibacterium* and Muribaculaceae in MOA group were observed in the cecum of piglets. Furthermore, the higher (*P* < 0.05) relative abundance of Actinobacteriota and Lactobacillaceae family in the Ctrl group, Ruminococcaceae and Lachnospiraceae in AGP group were noticed (Fig. 5L, M).

### Colonic microbiota

**Fig. 6:**
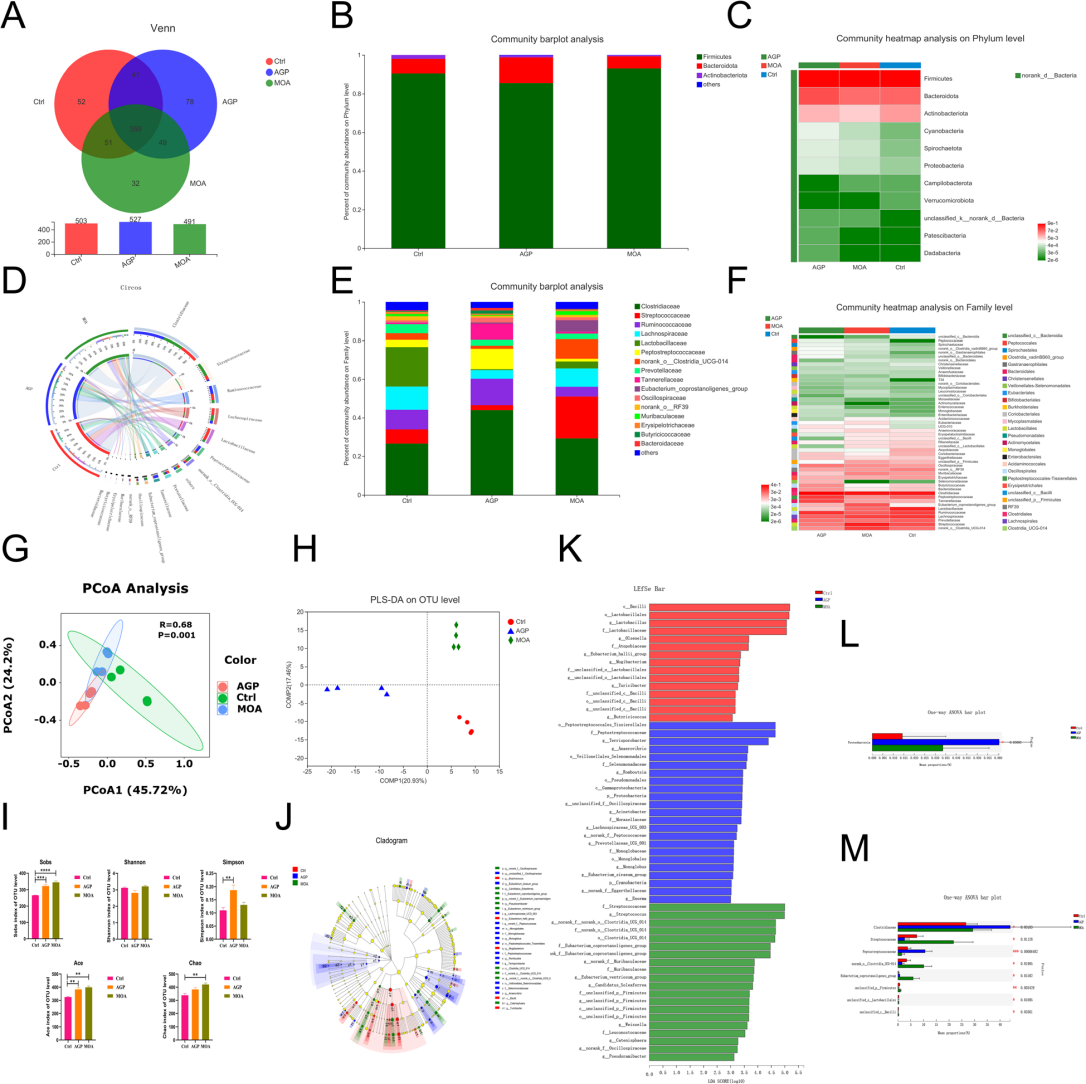
The microbial composition and structure of the colonic contents in piglets at 21 days of age as affected by dietary AGP and MOA supplementation. (A) Venn diagram. (B, E) Barplot analysis of microbial community compositions at phylum and family levels. (C, F) Heatmap analysis of microbial community compositions at phylum and family levels. (D) Circos diagram at family level. (G) Principal coordinate analysis (PCoA) based on bray-Curtis distance calculated from OTU abundance matrix (*R* = 0.68, *P* = 0.001). (H) Partial least squares discriminant analysis (PLS-DA) on OTU level, the analysis of similarities (ANOSIM) was used to exam the significant difference between treatments. (I) The α-diversity of microbial community, bar with the asterisk (*) level suggested the degree of significant difference and the values were indicated as means ± SEM (* 0.01 < *P* < 0.05, ** 0.001 < *P* < 0.01, *** 0.0001 < *P* < 0.001, **** *P* < 0.0001). (J) The discriminant analysis of LEfSe multi-level species difference from phylum to genus level. (K) Histogram of linear discriminant analysis (LDA) from phylum to genus level; the values were checked by a non-parametric factorial Kruskal-Wallis rank sum test to identify the microbes with the significant differential characteristics and a linear discriminant analysis was used to assess the degree of impact of abundance on the differences for each species. (L, M) Significance test of difference between treatments at phylum and family levels. Control (Ctrl): a corn soybean meal-based diet. AGP: Ctrl + 75 mg/kg chlortetracycline. MOA: Ctrl + 1500 mg/kg MOA. *n* = 4

From the Venn analysis (Fig. 6A) of OTU in cecum of piglets, which totally acquired 1521 OTU, and 359 were common OTU, the 52, 78 and 32 represented the unique OTU of Ctrl, AGP and MOA, respectively. At the phylum level (Fig. 6B, C), Firmicutes and Bacteroidetes were dominated phylum, which accounting in excess of 98%. The populations of Firmicutes in Ctrl, AGP and MOA group were 90.57%, 85.55% and 93.03%, respectively. The Bacteroidetes in Ctrl, AGP and MOA group were accounting for 7.45%, 13.23% and 6.32%, respectively. At the Family level (Fig. 6E, F), the microorganisms that dominated the top five in the Ctrl group were Clostridiaceae (26.59%), Lactobacillaceae (20.44%), Lachnospiraceae (12.04%), Ruminococcaceae (10.21%) and Streptococcaceae (7.35%). In the AGP group, primarily dominated by Clostridiaceae (43.95%), Ruminococcaceae (13.73%), PeptoStreptococcaceae (10.59%), Tannerellaceae (8.29%) and Lachnospiraceae (4.49%). In the MOA group, mainly dominated by Clostridiaceae (29.28%), Streptococcaceae (21.75%), norank_o_Clostridia UCG-014 (10.07%), Lachnospiraceae (9.51%) and Ruminococcaceae (5.05%). The Circos plots (Fig. 6D) reflect the proportional distribution of dominant species in colon of piglets among the treatments as well as the proportional distribution of each dominant species in the different treatments at the family level. From the PCoA based on Bray_curtis at OTU level (Fig. 6G), the contribution values of the two principal components PC1 and PC2 were PC1 = 45.72% and PC2 = 24.20%, respectively, and the treatments differ in microbial composition (*P* = 0.001, *R* = 0.68). Similar findings were acquired by the PLS-DA (Fig. 6H). The Sobs, Ace and Chao indices in colon of piglets supplemented with MOA were increased (*P* < 0.05). Also, dietary supplementation with AGP enhanced (*P* < 0.05) the Sobs, Simpson and Ace indices in colon of piglets (Fig. 6I). The findings (Fig. 6J, K) of LDA indicated that the colonic microbiota composition was affected by feeding modification. An enhanced richness of *Lactobacillus*, *Olsenella* and *Mogibacterium* in Ctrl group, *Terrisporobacter* and *Anaerovibrio* in AGP group as well as *Streptococcus*, *Weissella*, and Muribaculaceae in MOA group were noticed in the colon of piglets. Moreover, the higher (*P* < 0.05) relative abundance of Proteobacteria and PeptoStreptococcaceae and Clostridiaceae in the AGP group, Streptococcaceae and norank_o_Clostridia UCG-014 in MOA group were noticed (Fig. 6L, M).

## Discussion

Recently, with the rapid advancement of new feed additives as an alternative to antibiotics, the findings concerning the effects of mixed organic acids and essential oils on the growth performance and health status in piglets have been increasing [14, 28]. Also, the mechanisms and potential of organic acids and essential oils to replace antibiotics were elucidated in detail by Suiryanrayna and Ramana[29] and Zeng et al. [30], thus several studies were conducted on the effects of the combination of the two additives in weaned piglets [31, 32] and revealed that the combination of the essential oil and organic acid was superior to any single additive, which was in line with the results of the meta-analysis concerning antibiotic replacement products by Xu et al. [16]. Nonetheless, the powerful volatility of essential oils considerably weakens the antioxidant and antimicrobial effects of essential oils in weaned piglets [33]. Consequently, the present experiment combined the results of previous studies to address the volatility of essential oils by microencapsulation process, then comprehensively evaluated the effects of the combination of MOA in weaned piglets, including growth performance, immuno-antioxidant properties, digestive enzyme activity, intestinal morphology, and intestinal microbial structure.

Diarrhea in post-weaning is one of the major contributors to mortality and growth retardation in weaned piglets [1]. In the current study, no significant difference was occurred on ADG and ADFI of piglets, but a lowered diarrhea rate was observed in piglets supplementing of MOA from d 12 to 21, which was in agreement with the Tian et al. [34] and Long et al. [14], who indicated that dietary supplementation with the mixed essential oil and organic acid in piglets respectively, could reduce the diarrhea rate and increased the ADG of piglets. The mechanism might be associated with the mixed organic acids lowering the intestinal pH and inhibiting the proliferation of harmful bacteria in the intestine. Interestingly, the inconsistent result was documented by Yang et al. [35] and Xu et al. [36], who supplemented the mixture of essential oils and organic acids to diets of piglets and found that a positive effect on ADG, but no significant difference on diarrhea rate. It may be associated with the environment and the composition and proportions as well as the processing of essential oils and organic acids.

The immune antioxidant capacity of serum can be a reliable indicator of the health status of weaned piglets. IgG, IgA and IgM were the three necessary immunoglobulins to mirror the immune status of piglets [37]. The IL-10 is a cytokine, which could enhance the B-cell survival, proliferation and antibody production, block the NF-κB activity and reduce paracellular permeability, exerting the essential functions in immune regulation and inflammatory responses [38, 39]. In the current study, dietary supplementation with MOA enhanced the level of IL-10 on d 11 and have a tendency to increase the level of IgM and IL-10 on d 21, which was in accordance with our previous findings in broilers that dietary supplemented 6000 mg/kg of mixed organic acids increased the concentration of IgA and IL-10 [40]. Also, the similar result was observed in piglets by Pu et al. [41]. Mechanistically, organic acids increased the level of IL-10 primarily by differentially moderating Th1 and Th17 cell differentiation [42] or increase the concentration of IL-10 in T cells and regulatory B cells by inhibiting histone deacetylases and regulating the mammalian target of rapamycin [43, 44], thus improved the immune status in piglets.

After weaning, piglets are stimulated by factors such as environment and feed, causing disturbance of redox system of the body and a large accumulation of free radicals or reduced scavenging ability in piglets could lead to a decrease in feed intake and slow growth, especially affect smaller piglets more severely [45]. Therefore, increasing the level of antioxidant enzyme such as T-AOC, SOD, GSH-Px and reducing the level of serum MDA of piglets (an indicator reflecting the degree of tissue peroxidation) contributed to the alleviation of weaning stress [46]. In the current study, piglets fed MOA improved the level of serum T-AOC and GSH-Px on d 11 and enhanced the level of SOD in serum and GSH-Px in liver on d 21. The essential oils used in the present experiment were primarily thymol and eugenol, which have extremely powerful antioxidant capacity due to their phenolic hydroxyl groups, serving as hydrogen donors to peroxyl radicals in the first step of the oxidation reaction, thus effectively preventing and delaying lipid oxidation [47]. It was also confirmed by Tian et al. [34], who supplemented essential oil (13.5% thymol and 4.5% cinnamaldehyde) to the diets enhanced the level of serum SOD, T-AOC and catalase on d 14, lowered the content of MDA in serum and improved the level of GSH-Px in liver on d 28. Also, Xu et al. [36] supplemented MOA to the diets also reduced the level of serum MDA. Therefore, the improvement of the antioxidant properties in serum piglet was mainly attributed to the action of essential oils. The different types and concentrations of essential oils maybe the principal reasons for the differences in results.

The integrity of the morphological structure of the intestine is paramount for maintaining the normal intestinal function [48]. An atrophied intestinal villus or an elevated crypt depth demonstrated a reduced ability of the small intestine to absorb nutrients [49]. Additionally, lowered digestive enzyme activity was not conducive to nutrient absorption, causing reduced nutrient digestibility in piglets [50]. In the current study, dietary supplemented MOA enhanced the trypsin and lipase activities in pancreas compared to the AGP and improved the ATTD of DM, OM and GE in piglets. However, a beneficial effect on intestinal morphology in piglets dietary supplemented MOA was not noticed, which was similar with Xu et al. [36]. Mechanistically, mixed organic acids improved the absorption of nutrients in piglets by lowering the pH of the gastrointestinal tract to the optimum pH of pepsin (2.0–3.5), trypsin (7.8–8.5), and lipase (4–5.4) and increasing the activity of digestive enzymes [51]. However, one study suggested that the addition of essential oil and lactic acid to the diet did not notice any changes in digestive enzymes in the pancreas and small intestine of broilers [52], which might be connected with the low concentration of lactic acid, resulting in the inability to stimulate digestive enzyme activity. In addition, the improvement of intestinal morphology in piglets may be associated with the concentration of MOA products supplemented.

Weaning affects the number of intestinal bacteria, with a significant increase in the number of total aerobic bacteria, enterobacteria and enterococcus of piglets at the first week after weaning [53, 54]. The damage to the intestinal mucosa during weaning provided a substrate for pathogenic bacteria to reproduce, increasing the possibility of pathogenic bacteria adhesion and invasion. Also, the toxins and metabolites generated by pathogenic bacteria also destroyed the intestinal mucosal barrier (characterized by increased intestinal permeability), which affected the nutrient absorption and caused diarrhea in weaned piglets [55]. Hence, an intact intestinal mucosal barrier is crucial to guarantee the provision of adequate dietary nutrition to the whole body. The mechanical barrier, such as claudin-1, occludin, ZO-1, which regarded as principal constitution of tight junction and essential regulators in paracellular permeability [56], and chemical barriers, such as mucin-1, mucin-2, which were secreted by goblet cell and played crucial roles in modulating intestinal inflammation [57], were usually applied to assess the integrity of the intestinal barrier [58]. In the current study, dietary supplemented MOA showed a positive effect on the relative gene expression of claudin-1 and mucin-2 in ileum of piglets. Also, compared to the AGP, a beneficial effect in duodenum (claudin-1), jejunum (claudin-1, *ZO-1*, mucin-1) and ileum (occludin, claudin-1, mucin-2) were observed in piglets fed MOA, which was accordance with the findings by Pu et al. [41], who dietary supplemented 3000 mg/kg of benzoic acid, 400 mg/kg of *Bacillus coagulans* and 400 mg/kg of oregano oil improved the relative gene expression of claudin-1, occluding and mucin-2 in jejunum of piglets. It indicated that the enhanced nutrient digestibility and reduced diarrhea rates in piglets were probably associated with the improved intestinal mechanical and chemical barriers.

It is emerging that the intestinal microbiota occupied a decisive role in the physiological and health status of the host [59]. The mixed organic acids were considered to be effective ways to act in the distal intestine and regulate intestinal health, especially by altering the structure of bacterial cells through the action of essential oil, allowing organic acid to easily enter bacterial cell membranes and causing the death of some pathogenic bacteria [33]. Studies have documented that the higher the diversity and abundance of microorganisms, the stronger the organism’s ability to resist colonization by foreign pathogens. The diversity and abundance of intestinal microorganisms decreases when the organism is stressed [60]. In current research, dietary supplemented MOA enhanced the Sobs, Ace and Chao indices of microbiota in cecum, increased the Sobs, Ace and Chao indices of microorganism in colon, which was similar to our previous research [40]. Additionally, our study observed that dietary supplemented AGP increased the Simpson index of microorganisms in cecum of piglets, which was inconsistent with Yu et al. [61], who reported that AGP decreased the richness and diversity in ileum of suckling pigs. The differences occurred probably related to the type of antibiotic and the growth stage of the piglets. Nonetheless, Adewole et al. [62] and Ma et al. [37] reported that no significance difference was noticed on α-diversity in cecum of broilers. The probable reasons for this finding were attributed to the type of animal, age, diet composition and health status [63]. Similar to previous study in cecum of piglets [37], the Firmicutes and Bacteroidetes were dominated phylum.

Further analysis by β-diversity of microbiota, the microbial composition in the cecum and ileum of piglets differed between treatments, which was in agreement with the findings of Dai et al. [15] in broilers. *Faecalibacterium* was the paramount short chain fatty acid-producing bacterium in the human hindgut, also beneficial in improving diseases such as inflammatory bowel disease and colorectal cancer, which further explained the improvement of intestinal barriers and the increased concentration of volatile fatty acids (propionic acid and butyric acid) in the cecum [64]. Muribaculaceae are correlated with up-regulation of expression of genes related to carbohydrate metabolism [65]. *Streptococcus* were useful for reducing intestinal pathogen load [66]. *Weissella*, a species of lactic acid bacteria, were members of the autochthonous microorganism in livestock primarily, which was available as microbial products applied in direct feeding for livestock [67]. In addition, our present research noticed that dietary supplemented MOA increased the abundance of Streptococcaceae in colon of piglets, which include the essential species *Lactococcus lactis* (usually used in fermentative food) and the disease-causing *Streptococcus pneumoniae* [68], which was inconsistent with Xu et al. [32], who observed that dietary supplemented the essential oil and organic acid increased the abundance of *Lactobacillus* and Bacilli in the ileum after *ETEC F4* (K88^+^) challenge. The reason maybe relevant to differences in piglets (challenged or not) as well as intestinal segments (ileum or cecum). Yu et al. [61] founded that early antibiotic exposure changes the microbial composition of suckling piglets, leaving them in a vulnerable and unhealthy intestinal environment, which was also analogous to our research on piglets. Therefore, the improvement of microbial community in the cecum and colon of piglets contributed to the increased content of volatile fatty acids and modulated the relevant gene expression of intestinal barrier, improving intestinal health.

## Conclusions

In conclusion, dietary supplemented 1500 mg/kg of MOA alleviate diarrhea and improve nutrient apparent digestibility in piglets presumably by enhancing immuno-antioxidant properties, increasing digestive enzyme activity, up-regulating the expression of intestinal barrier-related genes, and modifying the microbial community structure of the cecum and colon. Compared to conventional single organic acid or essential oil products, MOA are capable of improving the health of piglets by avoiding absorption into the foregut segment and entering the hindgut to modulate the microbial structure. Nonetheless, the increased abundance of some potentially pathogenic bacteria (Streptococcaceae) could be attributed to the antimicrobial properties or concentration of the product, which remains to be further validated.

## Data Availability

Not applicable.

## Abbreviation

ADF: Acid detergent fiber
ADFI: Average daily feed intake
ADG: Average daily gain
AGP: Antibiotic growth promoters
ATTD: Apparent total tract digestibility
CD: Crypt depth
CP: Crude protein
DAO: Diamine oxidase
DLA: D-lactic acid
DM: Dry matter
EE: Ether extract
NDF: Neutral detergent fiber
FCR: Feed conversion ratio
GAPDH: Glyceraldehyde-3-phosphate dehydrogenase
GE: Gross energy
GSH-Px: Glutathione peroxidase
IFN-γ: Gamma-interferon
IgA, IgG, IgM: Immunoglobulins A, G, M
IL-1β: Interleukin-1β
IL-10: Interleukin-10
MDA: Malondialdehyde
MOA: Microencapsulated essential oils and organic acids
OM: Organic matter
OTU: Operational taxonomic unit
SOD: Superoxide dismutase
T-AOC: Total antioxidant capacity
TNF-α: Tumor necrosis factor-α
VH: Villi height
ZO-1: Zonula occludens-1
