# Correction: Microencapsulated essential oils combined with organic acids improves immune antioxidant capacity and intestinal barrier function as well as modulates the hindgut microbial community in piglets

**DOI:** 10.1186/s40104-024-01094-5

**Published:** 2024-09-27

**Authors:** Jiayu Ma, Shenfei Long, Jian Wang, Jie Gao, Xiangshu Piao

**Affiliations:** 1 State Key Laboratory of Animal Nutrition, College of Animal Science and Technology, China Agricultural University, Beijing, 100193 China; 2 Institute of Animal Sciences, Chinese Academy of Agricultural Sciences, Beijing, 100193 China

**Correction: J Animal Sci Biotechnol 13, 16 (2022)**

**https://doi.org/10.1186/s40104-021-00670-3**

Following publication of the original article [1], the authors reported an error in the MOA product and antibiotic section under Materials and Methods.

The original texts of this section was:

The product of MOA combination named PORCINATTM was supplied by Jefo (Jefagro, Canada), which is a selected formulation of essential oils primarily containing thymol, vanillin and eugenol and organic acid mainly containing fumaric, citric, butyric and sorbic acid microencapsulated in the triglyceride matrix of hydrogenated vegetable oils. The chlortetracycline was sourced from Tongli Xingke (Beijing Tonglixingke, China).

The correct texts should be:

The product of MOA combination named PORCINAT + ™ was supplied by Jefo Nutrition Inc. (Jefagro, Saint-Hyacinthe, Canada), which is a selected formulation of essential oils primarily containing thymol, vanillin and eugenol and organic acids mainly containing fumaric, sorbic, malic and citric acid microencapsulated in a triglyceride matrix of hydrogenated vegetable oils. The chlortetracycline was sourced from Beijing Tongli Xingke Agriculture Technology Co., Ltd. (Beijing, China).

The original article [1] has been updated.
